# Global agricultural productivity is threatened by increasing pollinator dependence without a parallel increase in crop diversification

**DOI:** 10.1111/gcb.14736

**Published:** 2019-07-10

**Authors:** Marcelo A. Aizen, Sebastián Aguiar, Jacobus C. Biesmeijer, Lucas A. Garibaldi, David W. Inouye, Chuleui Jung, Dino J. Martins, Rodrigo Medel, Carolina L. Morales, Hien Ngo, Anton Pauw, Robert J. Paxton, Agustín Sáez, Colleen L. Seymour

**Affiliations:** ^1^ Instituto Biodiversidad y Medio Ambiente (INIBIOMA) Universidad Nacional del Comahue‐CONICET San Carlos de Bariloche Rio Negro Argentina; ^2^ Instituto de Investigaciones Fisiológicas y Ecológicas Vinculadas a la Agricultura (IFEVA), Facultad de Agronomía Universidad de Buenos Aires‐CONICET Buenos Aires Argentina; ^3^ Naturalis Biodiversity Center Leiden The Netherlands; ^4^ Center for Environmental Sciences Leiden University Leiden The Netherlands; ^5^ Instituto de Investigaciones en Recursos Naturales, Agroecología y Desarrollo Rural (IRNAD) Universidad Nacional de Río Negro‐CONICET San Carlos de Bariloche Río Negro Argentina; ^6^ Department of Biology University of Maryland College Park Maryland; ^7^ Rocky Mountain Biological Laboratory Crested Butte Colorado; ^8^ Department of Plant Medicals Andong National University Andong Republic of Korea; ^9^ Mpala Research Centre and Department of Ecology & Evolutionary Biology Princeton University Princeton New Jersey; ^10^ Departamento de Ciencias Ecológicas, Facultad de Ciencias Universidad de Chile Santiago Chile; ^11^ Intergovernmental Science‐Policy Platform on Biodiversity and Ecosystem Services (IPBES) UN Campus Platz der Vereinten Nationen Bonn Germany; ^12^ Department of Botany and Zoology Stellenbosch University Matieland South Africa; ^13^ General Zoology, Institute for Biology Martin Luther University Halle‐Wittenberg Halle Germany; ^14^ German Centre for Integrative Biodiversity Research (iDiv) Halle‐Jena‐Leipzig Leipzig Germany; ^15^ Kirstenbosch Research Centre South African National Biodiversity Institute Claremont South Africa; ^16^ DST‐NRF Centre of Excellence, FitzPatrick Institute of African Ornithology University of Cape Town Rondebosch South Africa

**Keywords:** agricultural expansion, biodiversity, crop diversity, pollination, pollination services, pollinator‐dependent crops

## Abstract

The global increase in the proportion of land cultivated with pollinator‐dependent crops implies increased reliance on pollination services. Yet agricultural practices themselves can profoundly affect pollinator supply and pollination. Extensive monocultures are associated with a limited pollinator supply and reduced pollination, whereas agricultural diversification can enhance both. Therefore, areas where agricultural diversity has increased, or at least been maintained, may better sustain high and more stable productivity of pollinator‐dependent crops. Given that >80% of all crops depend, to varying extents, on insect pollination, a global increase in agricultural pollinator dependence over recent decades might have led to a concomitant increase in agricultural diversification. We evaluated whether an increase in the area of pollinator‐dependent crops has indeed been associated with an increase in agricultural diversity, measured here as crop diversity, at the global, regional, and country scales for the period 1961–2016. Globally, results show a relatively weak and decelerating rise in agricultural diversity over time that was largely decoupled from the strong and continually increasing trend in agricultural dependency on pollinators. At regional and country levels, there was no consistent relationship between temporal changes in pollinator dependence and crop diversification. Instead, our results show heterogeneous responses in which increasing pollinator dependence for some countries and regions has been associated with either an increase or a decrease in agricultural diversity. Particularly worrisome is a rapid expansion of pollinator‐dependent oilseed crops in several countries of the Americas and Asia that has resulted in a decrease in agricultural diversity. In these regions, reliance on pollinators is increasing, yet agricultural practices that undermine pollination services are expanding. Our analysis has thereby identified world regions of particular concern where environmentally damaging practices associated with large‐scale, industrial agriculture threaten key ecosystem services that underlie productivity, in addition to other benefits provided by biodiversity.

## Graphical abstract

Increasing cultivation of pollinator‐dependent crops has placed a stress on global pollination capacity, which could have been ameliorated by a concomitant increase in agricultural diversification. However, this study reports a relatively weak and decelerating rise in agricultural diversity over time that was largely decoupled from the strong and continually increasing trend in agricultural dependency on pollinators. Particularly worrisome is a rapid expansion of pollinator‐dependent monocultures in several countries of the Americas and Asia that has resulted in a decrease in agricultural diversity. In these regions, reliance on pollinators is increasing, yet agricultural practices that undermine pollination services are expanding.

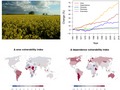

## 1 INTRODUCTION

Global agriculture has expanded at pace in recent decades, particularly in areas that formerly supported tropical and subtropical forests (Curtis, Slay, Harris, Tyukavina, & Hansen, 2018; Foley et al., 2011), and it has also become increasingly pollinator dependent (Aizen, Garibaldi, Cunningham, & Klein, 2008; Aizen & Harder, 2009). This latter trend can be attributed to the agricultural expansion of pollinator‐dependent crops, which include most oilseed, nut, and fruit crops, with a far lower rate of expansion of crops not dependent on pollinators, which include basic staple crops such as cereals (Aizen, Garibaldi, Cunningham, & Klein, 2009). However, one of the ultimate causes for the increase in agricultural pollinator dependency, a trend intensified with the acceleration of globalization in the early 1990s (Aizen & Harder, 2009), is the incentive provided by higher average market values for pollinator‐dependent crops (Gallai, Salles, Settele, & Vaissière, 2009; Lautenbach, Seppelt, Liebscher, & Dormann, 2012). A second, related cause is the lower intrinsic yield growth that characterizes most pollinator‐dependent crops, which often results in higher expansion rates to respond to growing market demands compared to nondependent crops (Aizen, Garibaldi, Cunningham, et al., 2009; Garibaldi, Aizen, Klein, Cunningham, & Harder, 2011). Despite the growth in demand for pollinator‐dependent crops, the availability of managed honeybees, the main commercial but not necessarily most efficient pollinator of many agricultural crops, has grown far slower than agricultural pollinator dependency (Aizen & Harder, 2009), and the picture is worse for wild pollinators that are in decline in several regions (e.g., Biesmeijer et al., 2006; Colla & Packer, 2008; Morales, Arbetman, Cameron, & Aizen, 2013; Ratto et al., 2018). As a consequence, the increased cultivation of pollinator‐dependent crops places a stress on global pollination capacity (Aizen & Harder, 2009).

While agricultural expansion itself is a major cause of pollinator decline through habitat loss and fragmentation and the use of pesticides and herbicides (De Palma et al., 2016; Kearns, Inouye, & Waser, 1998; Potts et al., 2010), threats to pollinators might be partially ameliorated by the cultivation of pollinator‐dependent crops that are sources of pollen and nectar (Deguines et al., 2014). However, although fields intensively cultivated with pollinator‐dependent crops can represent a cornucopia of food for both managed and wild bees and other pollinators, these fields may represent poor nesting habitats for most wild pollinators, and the food they provide may be available for only a short time (Garibaldi, Steffan‐Dewenter, et al., 2011; Rader et al., 2016; Westphal, Steffan‐Dewenter, & Tscharntke, 2003). As a consequence, a key issue from both ecological and economic perspectives is whether a trend toward more pollinator‐dependent agriculture has also fostered a more diversified agriculture, including the cultivation of more crops or greater representation of minor crops. This association is relevant because a more diversified agriculture has been linked to the maintenance of greater biodiversity in agroecosystems and with high‐quality ecosystem services that derive from it, a connection that has not only conservation but also important economic and social implications (Alho, 2008; Bhagwat, Willis, Birks, & Whittaker, 2008; Cardinale et al., 2012; Díaz et al., 2005; Haines‐Young & Potschin, 2005; Kremen & Merenlender, 2018). In particular, through increasing habitat heterogeneity and temporal availability of food resources, a more diverse agriculture can contribute to sustaining more diverse pollinator assemblages, and thus more efficient and stable pollination services (Garibaldi et al., 2013, 2014; Mandelik, Winfree, Neeson, & Kremen, 2016; Tscharntke, Klein, Kruess, Steffan‐Dewenter, & Thies, 2005).

Even though pollinator‐dependent crops account for less than one‐third of the total cultivated area and agricultural production, about 85% of the leading crop types, which may include one or several similar crop species (Klein et al., 2007), are, to varying extents, dependent on pollinators (Eardley et al., 2016). Given their expansion, one possibility is that a global increase in agricultural pollinator dependence over recent decades has led to an increase in agricultural diversification. Agricultural diversification, in terms of crop diversity, can involve both the global cultivation and commercialization of novel crops previously cultivated at regional and local scales, which would increase crop richness, and the expansion of commercially cultivated minor crops, which would increase crop evenness in terms of how total agriculture area is partitioned among different crops. An alternative possibility is that an increase in agricultural pollinator dependence has contributed little to agricultural diversification. This should be the case if the significant increase in global cultivated area observed over recent decades has been caused by the rapid expansion of large pollinator‐dependent monocultures, for example, oil palm or soybean, which today occupy vast agricultural areas and dominate the agriculture of several countries and entire regions (e.g., Aizen, Garibaldi, & Dondo, 2009; Lautenbach et al., 2012).

To evaluate these alternatives, we assessed changes in agricultural diversity in relation to agricultural expansion and increasing pollinator dependence between 1961 and 2016, using crop area data reported by countries to the Food and Agriculture Organization of the United Nations (FAOSTAT, 2018). Our assessment involved two levels of analysis. First, we described temporal trends in agricultural expansion, pollinator dependence of agriculture, and agricultural diversity, including both crop richness and evenness, at the global scale. Second, we analyzed the rates of change in agricultural diversity, including changes in both crop richness and evenness, in relation to rates of change in agricultural area and pollinator dependence at the country level, testing also for regional/continental differences in these trends. By addressing and comparing trends and rates of change in these variables at different geographic scales, our analysis provides not only a global assessment of the relation between agricultural diversity and increasing pollinator dependence but also identifies countries and regions where pollination services can be at risk due to a lack of an increase or even a reduction in agricultural diversification.

## 2 MATERIALS AND METHODS

The Food and Agriculture Organization of the United Nations (FAO) gathers annual information on crop cultivation (including area, production, and yield) at the global and country levels for 114 crops for which there is also information on pollinator dependence. Here, we focus exclusively on data for cultivated area (actually reported as harvested area) for all these crops from 1961 to 2016 (FAOSTAT, 2018). Crops included in our dataset collectively accounted for 95.6% and 94.3% of the total agriculture area in 1961 and 2016, respectively. Although most crops were represented by single species or, in a few instances, by varieties of the same species cultivated in different places, or harvested green or dry or for different parts, some were represented by a grouping of taxonomically related species (Aizen et al., 2008; Aizen, Garibaldi, Cunningham, et al., 2009; Klein et al., 2007; see Appendix S1). In this study, we followed FAO's original crop classification and considered each reported crop or crop item as a separate unit to minimize potential miscategorization.

Crops were characterized according to the extent to which biotic pollination contributes to their yield. We considered a crop to be pollinator dependent if animal pollination is required to increase the quantity and/or the quality of fruits or seeds directly consumed by humans. Alternatively, a crop was considered to be nondependent if it is pollinated either abiotically (wind) or autogamously (self‐fertilizing), or cultivated solely for vegetative parts (leaves, stems, tubers, etc.). This latter category includes crops like onions, potatoes, and other vegetables for which pollinators are not directly involved in the production of food, but are needed, in some cases, for the propagation of crops via seed or in breeding programs (see also Aizen et al., 2008; Aizen, Garibaldi, Cunningham, et al., 2009; Klein et al., 2007). Following Klein et al. (2007), crops were further classified into five classes of pollinator dependence based on the percent reduction in production (i.e., decreased fruit or seed set or weight) when pollinators are excluded experimentally from flowers. These include one nondependent category, “none” (i.e., no decrease in yield), and four dependent categories: “little” (yield reduction between >0% and ≤10%), “modest” (between >10% and ≤40%), “high” (between >40% and ≤90%), and “essential” (>90%). Although we reviewed more recent literature, we adopted the well‐accepted dependence values listed in Klein et al. (2007) to facilitate direct comparisons with other studies (e.g., Aizen, Garibaldi, Cunningham, et al., 2009; Lautenbach et al., 2012). The dataset of Klein et al. (2007) includes the most comprehensive compilation of pollinator‐dependence values available, even though these values probably underestimate real dependence as they are based on reports that, in almost all instances, do not consider the effects of varying pollinator abundance and assemblage composition on this estimation (e.g., Bartomeus et al., 2014; Ramos, Bustamante, da Silva e Silva, & Carvalheiro, 2018). We assigned pollinator‐dependent categories to 11 crops reported in the FAO dataset that were not previously classified by Klein et al. (2007) (Appendix S1). Because we aimed at connecting expansion in cultivation of pollinator‐dependent crops with agricultural diversification, we considered the proportion of the entire agricultural area cultivated with pollinator‐dependent crops as a measure of agricultural pollinator dependence, including in this group all crops from the “little” to “essential” categories (e.g., Aizen et al., 2008). Notwithstanding, we checked whether a differential areal expansion in cultivation occurred in crops of all dependent categories compared to those of the nondependent category.

Globally and for individual countries, we estimated the total agricultural area and agricultural pollinator dependence as the proportion of area under pollinator‐dependent crops, on a yearly basis from 1961 to 2016. We also estimated crop diversity as the effective number of crops $e^{H^{\prime }}$, where $H^{\prime }=-\sum p_{i}\cdot \ln\left(p_{i}\right)$, that is, Shannon–Wiener's index, and *p_i_* the proportion of total cultivated area accounted for by crop *i* of a total of *S* crops. Thus, the effective number of crops can be interpreted as the number of crops with the same cultivation areas that results in the observed *H*′ (see Jost, 2006). *H*′ incorporates both crop richness (i.e., number of different cultivated crops, *S*) and crop evenness (i.e., how total cultivated area is partitioned among different crops) as estimated by Pielou's index, *J*, where *J* = *H*′/ln(*S*). *J* varies from 0 to 1, approaching 0 when most area is devoted to the cultivation of just one crop and equaling 1 when all cultivated crops occupy equivalent area (Aizen, Garibaldi, & Dondo, 2009). Here, we analyzed crop diversity ($e^{H^{\prime }}$) and its two components, crop richness (*S*) and crop evenness (*J*). Although we refer to changes in crop diversity as trends in agricultural and crop diversification interchangeably, we recognize that agricultural diversification is a more encompassing term than crop diversification, as the former includes additional aspects related to land management and habitat heterogeneity (e.g., Sardiñas & Kremen, 2015; Sunderland & Samu, 2000) that are associated but not considered explicitly by the latter term.

In addition to assessing global temporal trends in total agricultural area, agricultural pollinator dependence, crop diversity, richness, and evenness, for each country, we estimated the average annual growth rate (%/year) for each of these variables (*x*) between 1961 and 2016 as $100\times \left(e^{\left[\frac{\ln\left(x_{2016}\right)-\ln\left(x_{1961}\right)}{2016-1961}\right]}-1\right)$ . Because several countries became politically subdivided after 1961, we combined the area cultivated for each crop during 2016 across the new countries when necessary. This was the case for the former Czechoslovakia (for which we added the crop figures of Czech Republic + Slovakia); Ethiopia PDR (Ethiopia + Eritrea); Sudan (South Sudan + Sudan); USSR (Azerbaijan + Belarus + Estonia + Georgia + Kazakhstan + Kyrgyzstan + Latvia + Lithuania + Republic of Moldova + Russian Federation + Tajikistan + Turkmenistan + Ukraine + Uzbekistan); and Yugoslavia SFR (Bosnia and Herzegovina + Croatia + Montenegro + Serbia + Slovenia + The former Yugoslav Republic of Macedonia). We also combined the data of Belgium and Luxembourg because agricultural data in 1961 were reported as Belgium‐Luxembourg in the FAO dataset. To explore geographical differences in growth rates, countries were grouped into one of the following geographical regions: Africa, America, Asia, Europe, and Oceania. The (ex) Union of Soviet Socialist Republics (USSR) was considered as part of Asia because most of its area belonged to that region. We excluded from this analysis all countries with an aggregate agricultural area <1,000 km^2^ in 1961 because some of them exhibited unusually high growth rates (i.e., outliers) in at least one variable. These anomalous values arose due to the small surface area devoted to agriculture in 1961, in some cases of only a few square kilometers, which added extremely high variance to evaluate general trends. Thus, our analysis of growth rates only included 127 of a total of 175 countries and former republics that account for ~99.9% of all cultivated land on earth in both 1961 and 2016. Because this criterion excluded most island states, this rule restricted our analysis for Oceania to only three countries (i.e., Australia, New Zealand, and Papua New Guinea).

We analyzed data on annual growth rates (Δ*s*) using main effect linear models (Zuur, Ineo, Walker, Saveliev, & Smith, 2009), with which we tested the effects of (a) geographical region (Region) on mean annual growth rate in agricultural area (Δ area); (b) Region and Δ area on annual growth rate in agricultural pollinator dependence (Δ dependence); and (c) Region, Δ area, and Δ dependence on annual growth rate in, alternatively, crop diversity (Δ diversity), crop richness (Δ richness), and crop evenness (Δ evenness). Because variances were suspected to differ among geographical regions, for each dependent variable we compared a model assuming homogeneous variances versus a model incorporating heterogeneous variance associated with this categorical variable (Zuur et al., 2009). These linear models were implemented using the *gls* function of package nlme (Pinheiro, Bates, DebRoy, & Sarkar, 2018) in R (R Core Team, 2018), assuming equal variances or, alternatively, modelling a unique variance for each level of the categorical variable (Region) by using the variance structure VarIdent. Pairwise Tukey's a posteriori tests were used to evaluate significant differences between regions using the *contrast* function of R's lsmeans package (Lenth, 2016).

Finally, we identified countries and regions where agricultural productivity and stability could be at risk due to growth in either agricultural area or pollinator dependence outpacing growth in agricultural diversification. For each of the 127 countries, we considered the differences between growth rates in area and diversity (i.e., Δ area − Δ diversity) and in pollinator dependence and diversity (i.e., Δ dependence − Δ diversity) as agricultural vulnerability indicators (see Birkmann, 2007). Therefore, values of these indices >0 indicate faster growth in agricultural area and pollinator dependence than diversity, respectively, and thus increasing risks to agricultural productivity through potential pollination shortfalls.

## 3 RESULTS

### 3.1 Global trends

**Figure 1:**
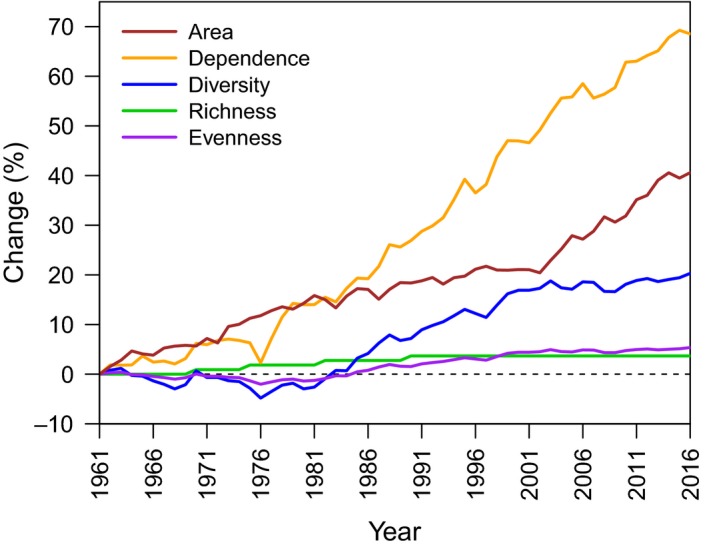
Global change in total land area devoted to agriculture, agricultural pollinator dependence (estimated as the proportion of total agricultural area occupied by pollinator‐dependent crops), crop diversity (estimated as the effective number of crops), crop richness (i.e., number of crops), and evenness (estimated as Pielou's index) between 1961 and 2016, based on crop area data in the FAO dataset (FAOSTAT, 2018). For each dependent variable, *x*, change from 1961 until year *t* is represented as a percentage of the value of *x* in 1961, that is, 100 (*x_t_* − *x*_1961_)/*x*_1961_

Globally, total agricultural area increased 40.6% from 1961 to 2016 (Figure 1), which in absolute terms represents an increase of 3.8 × 10^6^ km^2^ in cultivated land, including also cumulative area due to multiple harvests. The aggregate area cultivated with crops not dependent on pollinators increased by only 17.3%, whereas the area cultivated with pollinator‐dependent crops expanded by 136.9% (91.4%, 163.3%, 177.5%, and 117.7% for crops in the little, moderate, high, and essential categories of pollinator dependence, respectively; Figure S1). The global agricultural area occupied by pollinator‐dependent crops in 1961 was 19.4% but, because of their differential growth, by 2016, this percentage rose to 32.8% (Figure S1). As a consequence, the pollinator dependence of global agriculture, in terms of the proportion of area cultivated with pollinator‐dependent crops, increased ~70% from 1961 to 2016 (Figure 1).

The increase in pollinator dependence of global agriculture has been the consequence of rapid growth in the cultivated area of pollinator‐dependent crops (Appendix S1). Sixteen of the 20 (i.e., 80%) fastest expanding crops are, to varying degrees, pollinator dependent, whereas only six of the 20 (i.e., 30%) slowest expanding crops are pollinator dependent (*G*‐test of independence, *G*_1_ = 8.18, *p* = 0.004). On average, the area cultivated with pollinator‐dependent crops, in all four categories of pollinator dependence, expanded faster than that of nondependent crops (Figure S2). Despite not being included among the fastest expanding crops, because they were already cultivated over sizable areas in 1961 (Appendix S1), some pollinator‐dependent oilseed crops like soybean, canola, and oil palm (Figure S2) were responsible for much of the observed global agricultural expansion and increase in pollinator dependency.

Global crop diversity, as estimated by the effective number of crops, increased 20.5% between 1961 and 2016 (Figure 1). This increase was more closely related to changes in crop evenness than richness (*r* ^2^ = 0.998 vs. *r* ^2^ = 0.675, respectively). Indeed, at the global scale, only four of the 114 crops in the dataset were not present in 1961 (Appendix S1), and thus can be considered novel. Interestingly, crop diversity experienced an increase during the period 1980–2000, leveling off thereafter, at the same time that total agricultural area started to increase at a faster rate (Figure 1). Therefore, changes in crop diversity after 2000 were largely decoupled from changes in total agriculture area and in pollinator dependence of global agriculture, which has shown a steady increase since the late 1970s (Figure 1).

### 3.2 Regional and country‐level patterns

**Figure 2:**
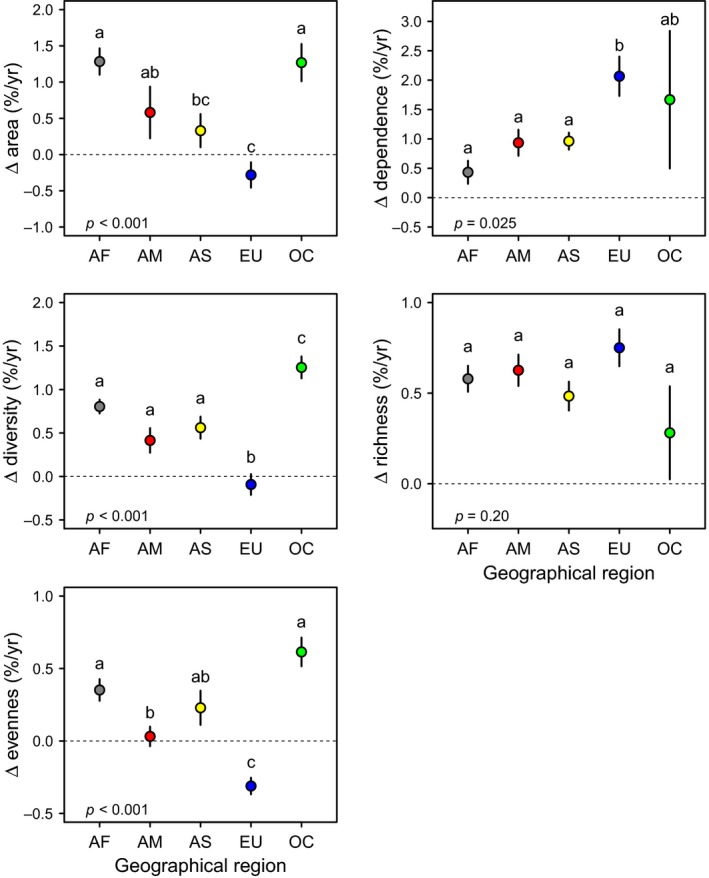
Least‐squares means (±1 *SE*) of growth in total agricultural area, agricultural pollinator dependence, and crop diversity, richness, and evenness for countries in each region of the world (i.e., AF, Africa; AM, America; AS, Asia; EU, Europe; OC, Oceania) between 1961 and 2016. Means with the same letter do not differ statistically at *α* = 0.05 according to a pairwise Tukey's a posteriori test. *p*‐Values correspond to the test for overall regional differences in each response variable (Table S1)

The relationship between the change in agricultural area and pollinator dependence was heterogeneous across regions and did not necessarily reflect the global pattern. For instance, African countries showed, on average, high rates of agricultural expansion but relatively low rates of increasing pollinator dependence (Figure 2). In contrast, European countries experienced a net contraction in agricultural area, but a marked increase in pollinator dependence (Figure 2). Countries in other regions of the world paralleled the global trend and exhibited positive and high growth rates of agricultural pollinator dependence with area expansion (Figure 2). Because of this heterogeneity, changes in agricultural area were not significantly related to changes in agricultural pollinator dependence at the country level (Figure S3), and different countries exhibited varying combinations of low and high growth rates in cultivated area and pollinator dependence (Appendix S2).

**Figure 3:**
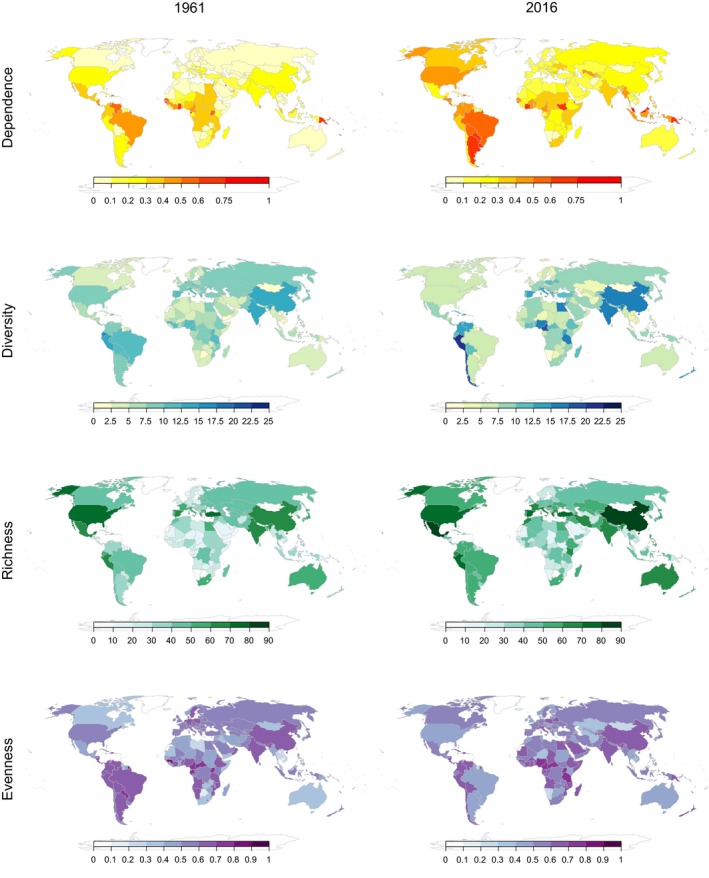
World maps of agriculture dependence on pollinators (i.e., the proportion of total cultivated area accounted for by pollinator‐dependent crops), crop diversity (estimated as the effective number of crops), crop richness (i.e., number of crops), and evenness (estimated as Pielou's index) in 1961 and 2016

**Figure 4:**
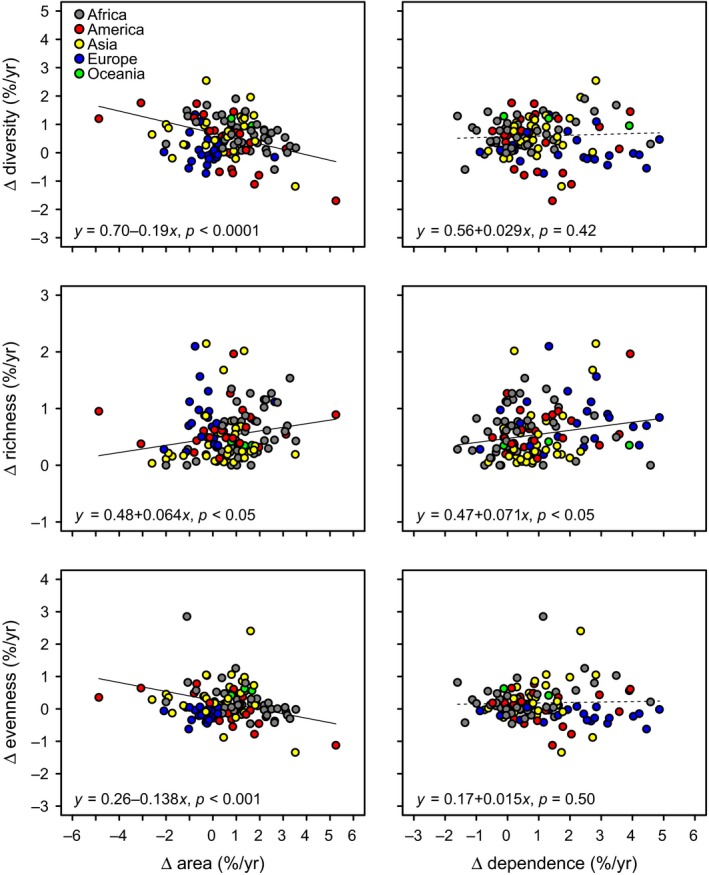
Mean annual growth rates in crop diversity, richness, and evenness in relation to yearly growth rates in total agricultural area (left panels) and agricultural pollinator dependence (right panels) for 127 countries. Solid and dashed lines indicate significant (*p* < 0.05) and nonsignificant (*p* > 0.05) partial regressions, respectively. *F*‐test statistics are provided in Table S1

In contrast to European countries, countries in other regions of the world exhibited, on average, increased crop diversification, with differences among regions mainly reflecting differences in crop evenness rather than crop richness (Figure 2). However, crop diversification was highly heterogeneous within regions. For instance, countries in South America's Pacific rim like Chile, Perú, and Colombia exhibited considerable increases in crop diversity, whereas important global agricultural producers and exporters on the Atlantic rim, like Argentina and Brazil, exhibited marked decreases in crop diversity (Figure 3; Appendix S2). Agriculturally important countries in Asia, like China and India, also showed remarkable increases in crop diversity (Figure 3; Appendix S2). Yet, at both regional and country levels, there was no evidence that increasing crop diversification resulted from either agricultural expansion or increasing cultivation of pollinator‐dependent crops. For instance, African countries exhibited increases in crop diversity comparable to countries from other regions, such as America and Asia, despite relatively low growth in agricultural pollinator dependence. In contrast, European countries did not show any increase in crop diversity despite notable increases in agricultural pollinator dependence, which reflects a replacement of nondependent crops by dependent crops (Figure 2). Although the cultivation of new crops was linked to both high rates of agricultural expansion and pollinator dependence at the country level, these variables were unrelated or even negatively related to agriculture diversification because of a negative or lack of influence on crop evenness, respectively (Figure 4). In particular, countries like Argentina, Brazil, USA, France, Germany, and Malaysia showed increased dependence on pollination but decreased agricultural diversity (Figure 3). Therefore, regional and country‐level analyses provide little support to the hypothesis that agricultural increasing pollinator dependence has fostered agricultural diversification.

**Figure 5:**
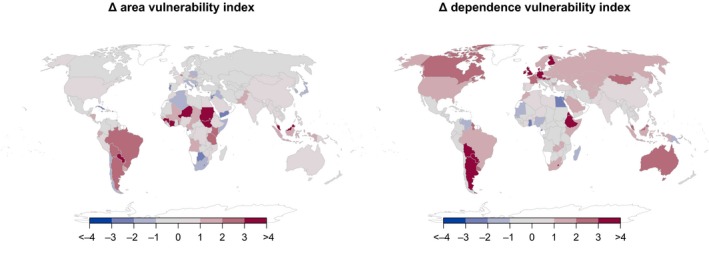
World maps of agricultural vulnerability through potential pollination shortfalls calculated as the difference between growth rates in agricultural area and crop diversity (Δ area vulnerability index) and between growth rates in agricultural pollinator dependence and crop diversity (Δ dependence vulnerability index) for 127 countries and former republics

Vulnerability indicators, which compare the potential negative effects of increasing agricultural area and increasing pollinator dependence with the positive effect of agricultural diversification, provided an integrative perspective of the observed geographical heterogeneity (Figure 5). Both vulnerability indicators, the index associated with expansion of agricultural area and the index associated with increasing agricultural pollinator dependence, were weakly but positively correlated at the country level (*r* = 0.188, *n* = 127, *p* = 0.033; Figure S4). Several African countries (most notably Niger, Guinea, Ivory Coast, and former Sudan) were particularly vulnerable because agricultural expansion largely outpaced agricultural diversification, whereas several European countries (most notably United Kingdom, Germany, France, Austria, Denmark, and Finland) and Australia were particularly vulnerable because increases in agricultural pollinator dependence largely outpaced agricultural diversification (Figure 5). However, South American countries like Argentina, Paraguay, Bolivia, and to a lesser extent Brazil and Uruguay, and Asian countries like Malaysia, and to a lesser extent Indonesia, were the most vulnerable of all because of both fast expansion in total agricultural area and pollinator dependence that were mostly associated with negative rates in agricultural diversification (Figure 5; Appendix S2).

## 4 DISCUSSION

Despite some fluctuations, global agriculture has become steadily more pollinator dependent at the global scale, particularly since the late 1970s. Here, we show that this trend has continued over the last decade since it was originally reported in 2008 (Aizen et al., 2008). This substantial increase might even be underestimated because, in the context of climate change, agriculture could become more pollinator dependent without any change in crop composition, as yield of heat‐stressed crops may be more reliant on outcross pollen (Bishop, Jones, Lukac, & Potts, 2016). Although agriculture became more diversified at the global scale between the early 1980s and late 1990s, our regional and country‐level analyses do not show evidence of an associated change in agricultural diversification with pollinator dependency. Therefore, at least in terms of crop diversity, our results do not support the hypothesis that an increase in pollinator dependence has promoted agricultural diversification at the global level.

### 4.1 Agricultural expansion, pollinator dependence, and crop diversification

Although most regions and countries exhibited positive rates of agricultural expansion and diversification (Figures 2 and 4), this diversification tended to occur at slower rates in countries that had undergone rapid expansion in agricultural area over the last five decades (Figure 4). Results indicate that the cultivation of new commercial crops could be related to a process of agricultural expansion. However, crop evenness, the most influential component of agricultural diversity, decreased with agricultural expansion at the country level, suggesting a trend toward increasing monoculture with increasing cultivated area (Figure 4). This is clearly exemplified by large North and South American countries such as the USA, Brazil, and Argentina, where the rapid expansion of moderately pollinator‐dependent oilseed crops like soybean (Aizen, Garibaldi, & Dondo, 2009; Fearnside, 2001; Ghosh & Jung, 2016; Johnston, 2014) has been associated with a decrease in agricultural diversity in recent decades (Figure 3). In Brazil, Argentina, Paraguay, and Bolivia, soybean expansion has been related to high rates of deforestation and biodiversity loss, so cultivation of this oilseed crop has come at a high environmental cost (Fearnside, 2001; Gasparri, Grau, & Gutiérrez Angonese, 2013; Grau, Gasparri, & Aide, 2005; Gudynas, 2008; Pacheco, 2006; Zak, Cabido, Cáceres, & Díaz, 2008). A similar scenario has played out in countries like Malaysia and Indonesia with the expansion of oil palm, another moderately pollinator‐dependent oilseed crop (Fayle et al., 2010; Fitzherbert et al., 2008; Koh & Wilcove, 2008). Therefore, in several agronomically important countries, agricultural expansion can be linked to monoculture over large areas of one or a few pollinator‐dependent crops, which caused a decrease in agricultural diversity at both landscape and country scales. Indeed, our vulnerability analysis identified these South American and Asian countries as those in which agricultural productivity and stability could be most at risk, because growth in both the area under agriculture and pollinator dependence of that agriculture largely outpaced any growth in agricultural diversification (Figure 5).

Despite the rising dominance of a few pollinator‐dependent crops, particularly oilseed crops that today occupy large agricultural areas, several less‐dominant pollinator‐dependent crops, particularly fruit and nut crops, are among the fastest expanding crops. This differential expansion has contributed substantially to agricultural diversification of countries located on the Pacific rim of South America (i.e., Chile, Perú, and Colombia). In contrast, the relatively high rates of agricultural diversification exhibited by several African countries (e.g., Egypt, Cameroon, NigeS2ria, Tanzania) were associated with no or only a weak increase in the pollinator dependence of their agriculture (Appendix ). The absence of a remarkable trend toward increased pollinator dependence in Africa (Figure 2) is likely due to the displacement of traditional staple crops such as millet and sorghum by other nonpollinator‐dependent crops such as maize (Pingali, 2017). In contrast to Africa, several European countries (e.g., France, Germany, Sweden, United Kingdom) showed increases in the pollinator dependence of their agriculture but little increase in agricultural diversity, because of the substitution of nondependent by pollinator‐dependent crops, particularly fruit crops (Lautenbach et al., 2012). These examples demonstrate heterogeneous regional and country‐level differences in how changes in the pollinator dependency of agriculture have affected, or not, the diversity of their agriculture.

### 4.2 Consequences and implications

The regional and country differences in the response of agriculture diversification to changes in agricultural pollinator dependence (Figures 2 and 3; Appendix S2) are likely to result in differences in their environmental, social, and economic consequences. Particularly worrisome is the observation that some countries underwent high rates of agricultural expansion and increase in agricultural pollinator dependence, concomitant with a decrease in agricultural diversity (Figures 4 and 5). This is the case for several South American countries mentioned above, which suffered high rates of deforestation for soybean expansion (Fehlenberg et al., 2017; Zak et al., 2008). Although there has been some debate about the extent to which pollinators contribute to soybean yield (Giannini, Cordeiro, Freitas, Saraiva, & Imperatriz‐Fonseca, 2015), some studies have reported that bees can increase soybean yield up to ~50% (Chiari et al., 2005; Milfont, Rocha, Lima, & Freitas, 2013; Zelaya, Chacoff, Aragón, & Blendinger, 2018). In this case, habitat loss along with increasing dominance of soybean in the agricultural landscape might jeopardize pollination services not only for soybean but also for other crops.

Habitat homogenization due to, for instance, monoculture expansion could be one of the most important drivers affecting bee abundance and diversity (Hendrickx et al., 2007; Kennedy et al., 2013; Quintero, Morales, & Aizen, 2009). For example, several crop pollination studies from Argentina report highly depauperate pollinator assemblages completely dominated by (often managed) honeybees (e.g., Chacoff & Aizen, 2005; Geslin et al., 2017; Sáez, Sabatino, & Aizen, 2012). High dependence of a country's agriculture on a single crop, particularly one that is pollinator dependent, increases a country's economic and food security vulnerability, not only because agricultural revenue is more subject to variable market values and climatic variability but also because of the instability in temporal yield associated with pollinator dependency (Garibaldi, Aizen, et al., 2011). In fact, yields of pollinator‐dependent crops are expected to become more variable and diminish when the abundance and diversity of wild bees decrease (Garibaldi et al., 2013). Therefore, where agricultural expansion is not accompanied by agricultural diversification, the risk of future pollination deficits may increase (Garibaldi et al., 2014). Those countries with a diversified agricultural sector benefit not only from the economic stability derived from lower production fluctuations (Liebman & Schulte, 2015) but also from the maintenance of more robust pollinator assemblages, particularly when their agricultural portfolio includes several pollinator‐dependent crops (Garibaldi et al., 2013, 2014; Mandelik et al., 2016; Tscharntke et al., 2005).

A key caveat of this study is that our large‐scale analyses could preclude downscale extrapolations. Scale issues are important because a positive effect of agricultural diversification (or the lack of it) on ecosystem services, like pollination, has been mostly documented at local rather than larger scales (e.g., Carvalheiro, Seymour, Nicolson, & Veldtman, 2012; Holzschuh, Dormann, Tscharntke, & Steffan‐Dewenter, 2011; Kremen & Miles, 2012). Nevertheless, changes in local agricultural diversification that are consistent over extensive areas should scale up at the country and regional levels. In particular, changes in agricultural diversity at large spatial scales may result from local‐scale, environmentally friendly agriculture schemes, such as crop rotation and intercropping that could enhance different ecosystem services, particularly pollination (reviewed in Garibaldi et al., 2014). Alternatively, a country could have a diversifying agricultural trend driven by increasing local monocultures, with crops differing among areas within the country. Although a local increase in crop diversity could be a minor component of a country‐ or regional‐level trend, large‐scale agriculture diversification could still be relevant for ecosystem services. For example, a recent study has shown that bee beta diversity (i.e., bee species turnover) at a scale of thousands of square kilometers can be a key component of efficient pollination service, because different dominant and less abundant bee species are needed to provide the same service in different areas (Winfree et al., 2018). Also, different crops cultivated in different areas might increase the phenological match between flower and pollinator availability, particularly under a scenario of climate change (Bellard, Bertelsmeier, Leadley, Thuiller, & Courchamp, 2012). Therefore, biodiversity in general, and agriculture diversity in particular, may foster more efficient pollination services across any spatial scale (Deguines et al., 2014).

## 5 CONCLUDING REMARKS

Although our analysis reveals a weak increase in global agricultural diversity over recent decades, this trend has not kept pace with the marked increase in total cultivated area (Schmitz et al., 2014), or with the proportion of land devoted to the cultivation of pollinator‐dependent crops (Aizen et al., 2008). More specifically, our results indicate that the differential expansion in cultivation of several pollinator‐dependent crops observed recently has not contributed substantially to a more diversified agriculture globally. Therefore, if the current trend of an increasingly pollinator‐dependent agriculture continues, there will be an increasing global demand for pollination services and risk of pollination shortfall caused by reduced biodiversity, a by‐product of a less diversified agriculture. However, the consequences of this trend at the regional and country levels could be quite heterogeneous. In particular, our analysis provides a useful approach to identify countries and regions that are particularly vulnerable because the steep increase in their agricultural pollinator dependence has come about at a potentially high environmental cost, and may not be compensated by the economic and social benefits associated with more diverse agricultural practices. Therefore, an increase in agricultural pollinator dependency without a parallel increase in agricultural diversification is an alarm call for the implementation of more pollinator‐friendly, synergistic management, including targeted use of insecticides, the setting aside of marginal land to establish and maintain flower strips and hedgerows, and the restoration of seminatural and natural areas adjacent to crops (Garibaldi et al., 2014). Such changes, in addition to increasing crop diversity at different spatial scales, will increase farmland heterogeneity, fostering pollination services and thus agricultural productivity and sustainability.
