# Supplementary material for: Global agricultural productivity is threatened by increasing pollinator dependence without a parallel increase in crop diversification
Source: Glob Chang Biol. 2019 Jul 10;25(10):3516–27. doi: 10.1111/gcb.14736 (PMC6852307; doi:10.1111/gcb.14736)
Supplement: Supplementary file 3 [file GCB-25-3516-s003.docx]

**SUPPORTING INFORMATION**

**Global agricultural productivity is threatened by increasing pollinator dependence without a parallel increase in crop diversification**

Marcelo A. Aizen^1^| Sebastián Aguiar^2^| Jacobus C. Biesmeijer^3,4^| Lucas A. Garibaldi^5^| David W. Inouye^6,7^| Chuleui Jung^8^| Dino J. Martins^9^| Rodrigo Medel^10^| Carolina L. Morales^1^| Hien Ngo^11^| Anton Pauw ^12^| Robert Paxton^13,14^| Agustín Sáez^1^ | Colleen Seymour^15,16^

^1^Instituto Biodiversidad y Medio Ambiente (INIBIOMA), Universidad Nacional del Comahue-CONICET, San Carlos de Bariloche, Rio Negro, Argentina

^2^Instituto de Investigaciones Fisiológicas y Ecológicas Vinculadas a la Agricultura (IFEVA), Universidad de Buenos Aires-CONICET, Facultad de Agronomía, Buenos Aires, Argentina.

^3^Naturalis Biodiversity Center, Leiden, The Netherlands

^4^ Center for Environmental Sciences, Leiden University, The Netherlands

^5^Instituto de Investigaciones en Recursos Naturales, Agroecología y Desarrollo Rural (IRNAD), Universidad Nacional de Río Negro-CONICET, San Carlos de Bariloche, Río Negro, Argentina

^6^Department of Biology, University of Maryland, College Park, Maryland

^7^Rocky Mountain Biological Laboratory, Crested Butte, Colorado

^8^Department of Plant Medicals, Andong National University, Andong, Republic of Korea

^9^ Mpala Research Centre and Department of Ecology & Evolutionary Biology, Princeton University, Princeton, New Jersey

^10^Departamento de Ciencias Ecológicas, Facultad de Ciencias, Universidad de Chile, Santiago, Chile

^11^Intergovernmental Science-Policy Platform on Biodiversity and Ecosystem Services (IPBES), UN Campus Platz der Vereinten Nationen, Bonn, Germany

^12^ Department of Botany and Zoology, Stellenbosch University, Matieland, South Africa

^13^General Zoology, Institute for Biology, Martin Luther University Halle-Wittenberg, Halle, Germany

^14^German Centre for Integrative Biodiversity Research (iDiv) Halle-Jena-Leipzig, Leipzig, Germany

^15^South African National Biodiversity Institute, Kirstenbosch Research Centre, Claremont, South Africa

^16^FitzPatrick Institute of African Ornithology, DST-NRF Centre of Excellence, University of Cape Town, Rondebosch, South Africa

**SUPPORTING INFORMATION CAPTIONS**

**Figure S1** Global temporal trends in total agricultural area accounted for by crops that do not depend on pollinators (none) and crops that depend to different extents on pollinators based on the percentage of yield reduction in the absence of pollinators (little: between >0 and <10%; modest: between >10 and <40%, high: between >40 and <90 %; and essential: >90%).

**Figure S2** Mean annual rate of expansion in area (%/yr) between 1961-2016 for 110 crops ordered according to increasing rate of expansion. Crops are classified according to their pollinator dependence based on the percentage yield reduction in the absence of pollinators (none: 0%; little: 0-10%; modest: 10-40%, high: 40-90 %; and essential: >90%). The mean (+ 1SE) for each dependence category is shown in the panel below. According to a one-way ANOVA, means for the different pollinator-dependent categories differ significantly in the rate of expansion of crop area (*F*_4,105_ = 3.67, *p*=0.0082), among which an a priori orthogonal contrast shows significant differences between the non-dependent and pollinator-dependent categories (*F*_1,105_ = 8.08, *p*=0.007).

**Figure S3** Annual growth rates in agricultural pollinator dependence in relation to annual growth rates in total agricultural area for 127 countries. The dashed line depicts a non-significant linear regression. *F*-test statistics are provided in Table S1.

**Figure S4** Scatter plot of agricultural vulnerability indices calculated as the differences between the growth rates in agricultural area and crop diversity (Δ area vulnerability index) and between the growth rates in agricultural pollinator dependence and crop diversity (Δ dependence vulnerability index) for 127 countries. The zero reference value for each of the two variables is indicated with a dotted line.

**Table S1**. Main-effect linear models testing the effects of geographical region (Region) on mean annual growth rate in agricultural area (Δ area); Region and Δ area on annual growth rate in agricultural pollinator dependence (Δ dependence); and Region, Δ area and Δ dependence on annual growth rate in, alternatively, crop diversity (Δ diversity), crop richness (Δ richness), and crop evenness (Δ evenness). Mean annual growth rates (%/ yr) between 1961 and 2016 were estimated for 127 countries with >1000 km^2^ of agriculture area in 1961. Data from countries that split after 1961 (e.g. USSR) were combined (see Materials and Methods). For each dependent variable, the result of a (log) likelihood ratio test comparing the model that assumes homogeneous variances and the model incorporating heterogeneous variances in the categorical predictor (i.e., Region) is provided, if significant heterogeneity in variances was detected. *F*- and *p*-values correspond to the model with the best fit. *p*-values < 0.05 for individual predictors are boldfaced.


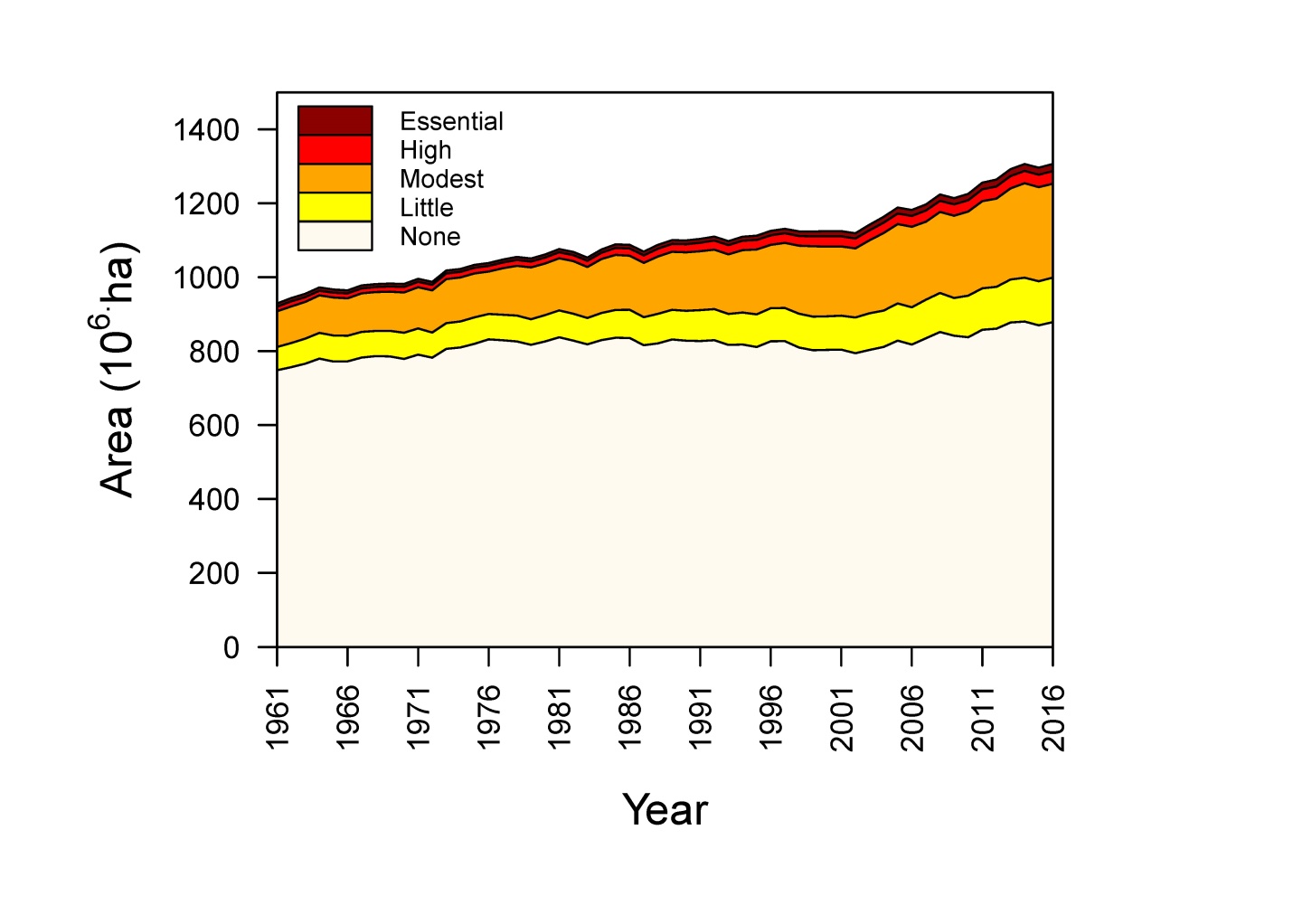


**Figure S1** Global temporal trends in total agricultural area accounted for by crops that do not depend on pollinators (none) and crops that depend to different extents on pollinators based on the percentage of yield reduction in the absence of pollinators (little: between >0 and <10%; modest: between >10 and <40%, high: between >40 and <90 %; and essential: >90%).


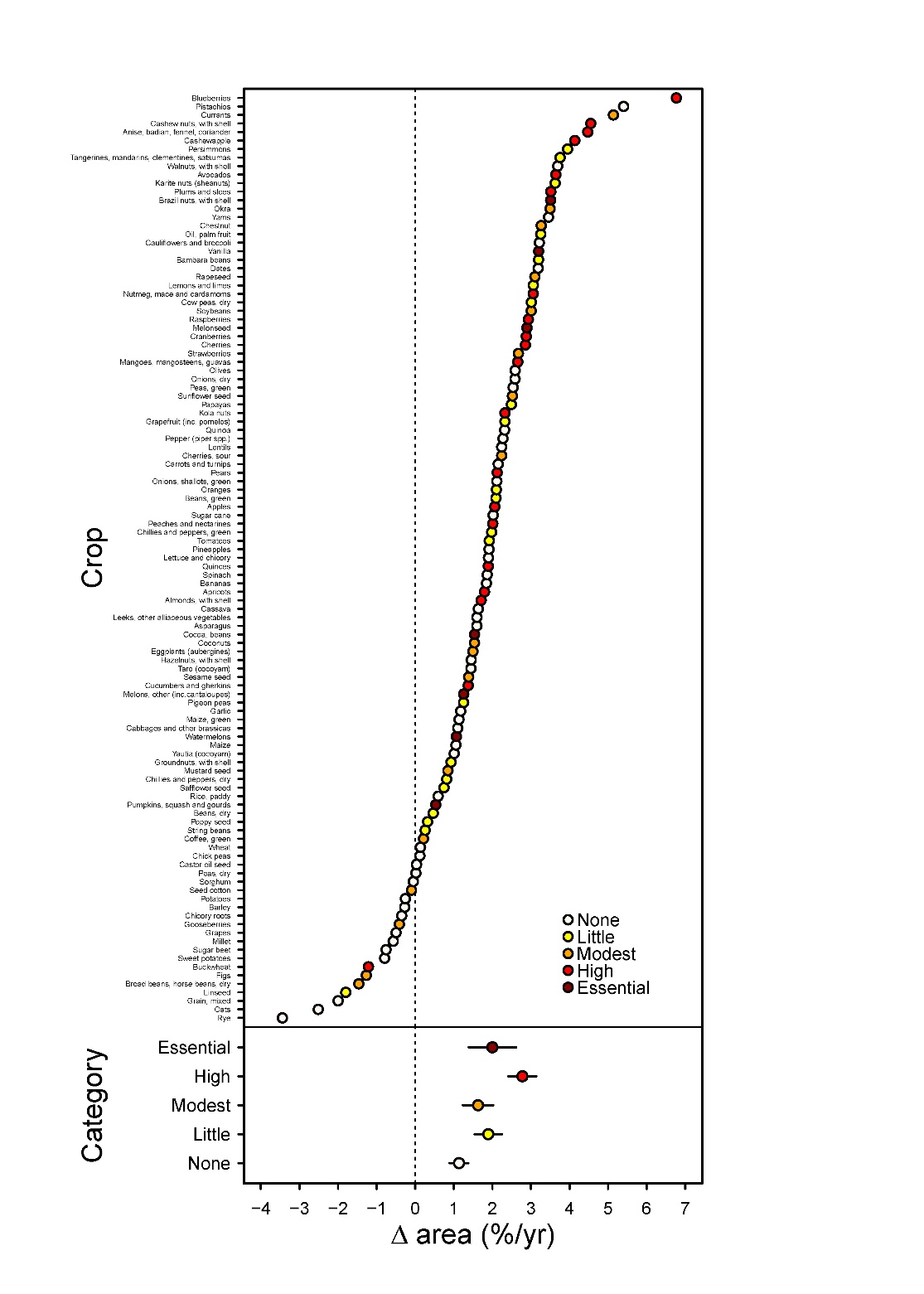


**Figure S2** Mean annual rate of expansion in area (%/yr) between 1961-2016 for 110 crops ordered according to increasing rate of expansion. Crops are classified according to their pollinator dependence based on the percentage yield reduction in the absence of pollinators (none: 0%; little: 0-10%; modest: 10-40%, high: 40-90 %; and essential: >90%). The mean (+ 1SE) for each dependence category is shown in the panel below. According to a one-way ANOVA, means for the different pollinator-dependent categories differ significantly in the rate of expansion of crop area (*F*_4,105_ = 3.67, *p*=0.0082), among which an a priori orthogonal contrast shows significant differences between the non-dependent and pollinator-dependent categories (*F*_1,105_ = 8.08, *p*=0.007).


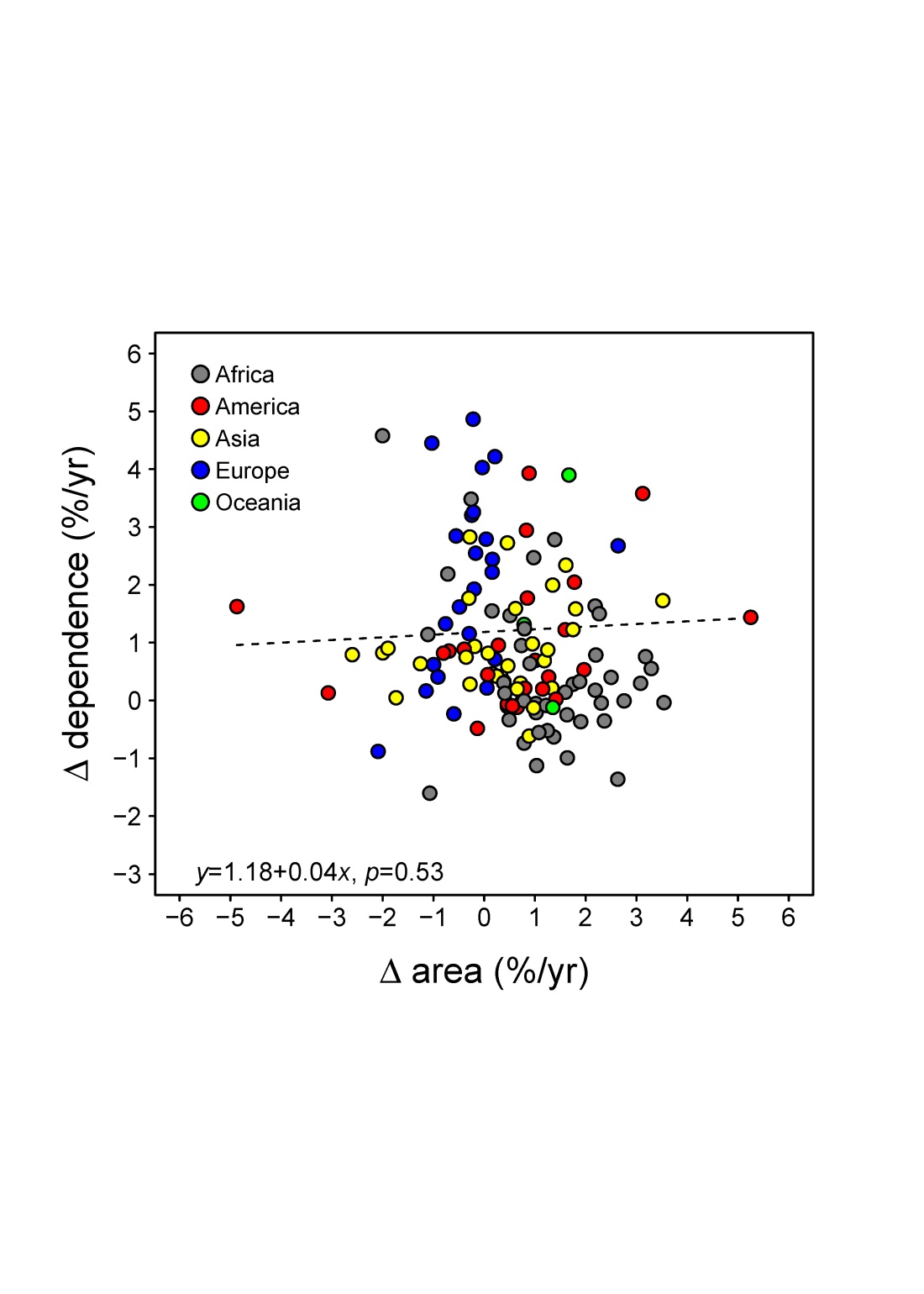


**Figure S3** Annual growth rates in agricultural pollinator dependence in relation to annual growth rates in total agricultural area for 127 countries. The dashed line depicts a non-significant linear regression. *F*-test statistics are provided in Table S1.


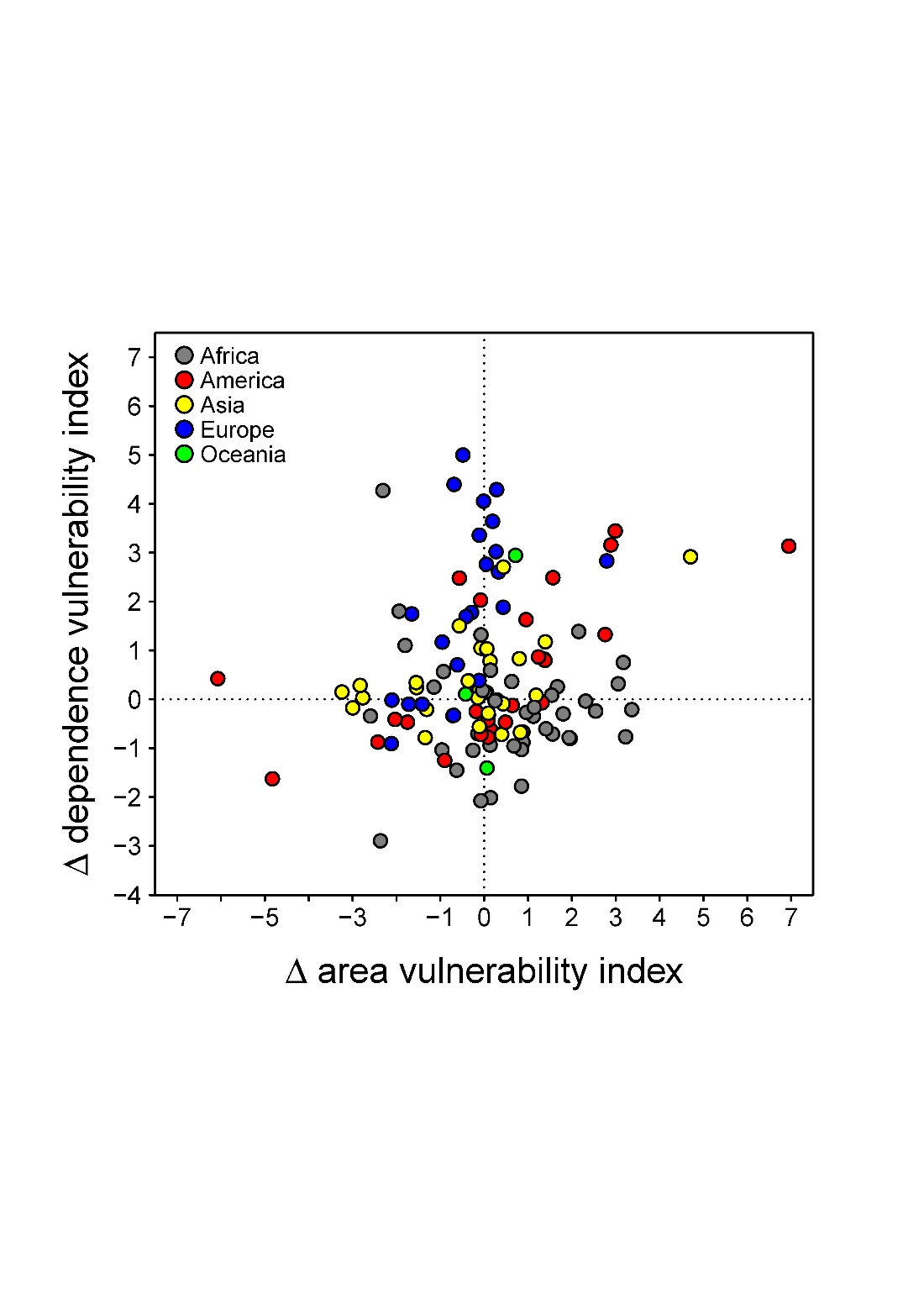


**Figure S4** Scatter plot of agricultural vulnerability indices calculated as the differences between the growth rates in agricultural area and crop diversity (Δ area vulnerability index) and between the growth rates in agricultural pollinator dependence and crop diversity (Δ dependence vulnerability index) for 127 countries. The zero reference value for each of the two variables is indicated with a dotted line.

**Table S1** Main-effect linear models testing the effects of geographical region (Region) on mean annual growth rate in agricultural area (Δ area); Region and Δ area on annual growth rate in agricultural pollinator dependence (Δ dependence); and Region, Δ area and Δ dependence on annual growth rate in, alternatively, crop diversity (Δ diversity), crop richness (Δ richness), and crop evenness (Δ evenness). Mean annual growth rates (%/ yr) between 1961 and 2016 were estimated for 127 countries with >1000 km^2^ of agriculture area in 1961. Data from countries that split after 1961 (e.g. USSR) were combined (see Materials and Methods). For each dependent variable, the result of a (log) likelihood ratio test comparing the model that assumes homogeneous variances and the model incorporating heterogeneous variances in the categorical predictor (i.e., Region) is provided, if significant heterogeneity in variances was detected. *F*- and *p*-values correspond to the model with the best fit. *p*‑values < 0.05 for individual predictors are boldfaced.

|  |  | Δ area | |  | Δ dependence | |  | Δ diversity | |  | | Δ richness | |  | Δ evenness | |
| --- | --- | --- | --- | --- | --- | --- | --- | --- | --- | --- | --- | --- | --- | --- | --- | --- |
| Predictor | ndf | *F*_ndf,118_ | *p* |  | *F*_ndf,117_ | *p* |  | *F*_ndf,116_ | *p* | |  | *F*_ndf,120_ | *p* |  | *F*_ndf,116_ | *p* |
| Δ area | 1 | _ | _ |  | 0.39 | 0.53 |  | 22.10 | **<0.001** | |  | 4.36 | **0.039** |  | 26.60 | **<0.001** |
| Δ dependence | 1 | _ | _ |  | _ | _ |  | 0.67 | 0.42 | |  | 4.76 | **0.031** |  | 0.45 | 0.50 |
| Region | 4 | 11.79 | **<0.001** |  | 4.36 | **0.025** |  | 15.84 | **<0.001** | |  | 1.51 | 0.20 |  | 20.56 | **<0.001** |
|  |  | MS_error_ = 1.278 | |  | MS_error_=0.803 | |  | MS_error_=0.706 | |  | | MS_error_=0.442 | |  | MS_error_= 0.657 | |
| Likelihood ratio test | | χ^2^ =16.46 | |  | χ^2^ =13.42 | |  | χ^2^ =9.81 | |  | | _ | |  | χ^2^ =29.72 | |
